# Computational Approach to the Surface-Crosslinking Process of Superabsorbent Polymer via Central Composite Design

**DOI:** 10.3390/polym14183842

**Published:** 2022-09-14

**Authors:** Hae-Chan Kim, Yong-Rok Kwon, Jung-Soo Kim, Miyeon Kwon, Jong-Ho Kim, Dong-Hyun Kim

**Affiliations:** 1 Material & Component Convergence R&D Department, Korea Institute of Industrial Technology (KITECH), Ansan 15588, Korea; 2 Department of Materials Science and Chemical Engineering, Hanyang University, Ansan 15588, Korea

**Keywords:** superabsorbent polymer composites, surface-crosslinking, central composite design, response surface methodology, absorption property

## Abstract

The improvement of gel strength and absorption properties through the surface-crosslinking of superabsorbent polymers (SAPs) is essential for sanitary industry applications. We prepared core-SAP via aqueous solution copolymerization, and then surface-crosslinked the core-SAP under various conditions. The structure of the SAP was characterized using Fourier transform infrared (FT-IR) spectroscopy. Central composite design (CCD) of response surface methodology (RSM) has been applied to determine the optimum surface-crosslinking conditions such as surface-crosslinker content, reaction temperature, and reaction time. The optimal surface-crosslinking conditions were identified at a surface-crosslinker content of 2.22 mol%, reaction temperature of 160 °C, and reaction time of 8.7 min. The surface-crosslinked SAP showed excellent absorbency under load of 50 g/g with a permeability of 50 s. Other absorption properties were also evaluated by measuring the free absorbency and centrifuge retention capacity in saline solution.

## 1. Introduction

Superabsorbent polymers (SAPs) are hydrophilic polymeric hydrogels with a three-dimensional network structure that can absorb a large amount of water [1]. SAPs in the sanitary industry demand better absorption properties because the SAP-containing products must be lightweight and small in volume [1,2]. In a disposable diaper, the occurrence of pressure due to the wearer’s physical activity intrinsically causes the absorbed water to be problematically discharged due to low SAP gel strength [1,2,3]. To address this, SAP research has focused recently on improving gel strength. The core–shell structure of SAPs through surface-crosslinking was designed to absorb a large amount of water. Furthermore, SAPs have the potential to show high gel strength and excellent absorption characteristics under pressure [4,5].

The majority of existing commercial SAPs are composed of acrylic acid (AA) and acrylamide, both of which are derived from petroleum. Because the use of substances derived from petrochemicals can hinder the present global efforts towards carbon neutrality, it is necessary either to seek alternatives or to reduce the number of petrochemicals in use [6]. Research on eco-friendly SAPs has been performed utilizing accessible biomass-derived materials (e.g., starch and cellulose) that are not harmful to the human body [7]. Synthetic routes include the graft copolymerization of vinyl monomers onto natural polymer chains to generate SAPs based on carboxymethyl cellulose [8], chitosan obtained by the deacetylation of chitin from the shells of crab, crayfish, and shrimp [9], and the use of starch [10]. Although these natural polymers were selected for their absorption properties, the natural polymer-based SAPs have a limited capacity for spatial chain expansion compared to traditional petroleum-based SAPs [11,12].

Itaconic acid (IA) is listed among the top value-added chemicals in biomass according to the US Department of Energy, and is presently regarded as a potential alternative to acrylic acid as the main raw material for SAPs [13]. However, the absorption performance of IA-based SAPs is limited by their low gel strength [14,15].

Central composite design (CCD) is the most widely used response surface methodology (RSM) approach. Utilizing RSM-CCD application has several advantages, such as experiment accuracy, minimizing experimental attempts, and the amount of information obtained for measuring the fit, unlike conventional experimental methods conducted without any clear plan [16].

In order to develop or produce a better quality product, design and experimentation are required to improve product performance. There may also be problems with finding optimal results at a limited cost. RSM is a statistical approach that can increase experimental efficiency and find optimal results while minimizing experimentation. CCD can identify factors affecting optimization and product quality in an experiment that considers multiple response factors, while also determining the level of factors to derive optimal results.

In this study, we attempted to develop biomass-derived IA-based SAPs with improved gel strength using RSM-CCD modeling. To the best of our knowledge, there has been no study to establish surface-crosslinking conditions applying RSM modeling in an itaconic acid-based SAP. In previous studies on surface-crosslinking in the SAP industry, there was no clear surface-crosslinking process applicable to CSAPs due to differences in their mechanical and absorption properties. In addition, it is difficult to distinguish the exact critical point of their absorption performance results by setting numerous existing random experimental groups. Therefore, the use of RSM-CCD modeling techniques is a more precise scientific optimization method that can respond to various variables. The utilization of this RSM-CCD modeling technique in various CSAPs through many studies will help establish their optimal surface-crosslinking process.

## 2. Materials and Methods

### 2.1. Materials

Itaconic acid (IA, ≥99%, Junsei Chemical, Tokyo, Japan) and acrylic acid (AA, 99%, Sigma Aldrich, St. Louis, MI, USA) were used as received without further purification. Aqueous sodium hydroxide solution (50%, Samchun Chemicals, Seoul, Korea) was used as a neutralizing agent. Ammonium persulfate (APS, reagent grade, 98%, Sigma Aldrich) was used as the initiator. The inner-crosslinker required for polymerization was crosslinked using 1,6-hexanediol diacrylate (HDODA, 99%, Sigma Aldrich), and then 1,4-butanediol (BD, 99%, Sigma Aldrich) was used as a surface-crosslinker. Methanol (MeOH, 99.5%, SamchunChemicals) was used as a medium in the surface-crosslinking solution. All reagents used in the experiment were used in a purchased state without a separate purification process.

### 2.2. Preparation of Core-SAP

The initiator APS decomposed from the heat source to produce radicals, and partially neutralized poly(IA-co-AA) was synthesized by applying aqueous solution polymerization through radical transition and chain growth of vinyl groups of IA and AA [17]. The polymerization reactions for the preparation of core-SAP (CSAP) were performed in a 500 mL four-necked flat bottom flask. The flask was fastened with a mechanical stirrer, a reflux condenser, a thermometer, and a needle for the injection of nitrogen gas. First, IA and AA were mixed at a molar ratio of 0.38:0.55 in a set amount of 50% NaOH solution and then stirred at 250 rpm through a mechanical stirrer for 30 min at 60 °C. This was followed by the addition of APS (0.5 wt%) and HDODA (2.0 wt%). After stirring for 1 h, the mixture was taken for gelation in a convection oven at 60 °C. After completion of gelation, the particles were pulverized into 300–600 μm particles. Finally, the CSAP particles were washed in excess acetone to remove any unreacted monomers, oligomers, and inner-crosslinker.

### 2.3. Fourier Transform Infrared Spectroscopy Characterization

The structures of the SAPs (in the form of potassium bromide pellets) were examined via Fourier transform infrared spectroscopy (FT-IR, NEXUS Instruments, Thermo Nicolet NEXUS 670, Donaueschingen, Germany) using a scan range of 500–4000 cm^−1^ at a resolution of 1 cm^−1^.

### 2.4. Central Composite Design

The study on the surface-crosslinking process was conducted using CCD with four factors (surface-crosslinker content, solution immersion time of core-SAP, reaction temperature, and reaction time). Minitab software (Minitab Inc, State College, PA, USA) was used to design a set of 20 experiments with varying values of the selected factors. In this study, the response (absorption capacity) was estimated for all required experimental trials. The coded patterns in the design represent scaled factor values, coded as (−1, 0, +1). Table 1 shows the design values by CCD of 3 factors. For each experimental group derived via CCD, the average value was applied through a total of 3 replicates.

### 2.5. Centrifuge Retention Capacity

The centrifuge retention capacity (CRC) is an absorption characteristic that measures the amount of fluid SAP retains after dehydration through centrifugation of freely absorbed SAP (swollen SAP in the absence of pressure). The CRC test method was measured according to EDANA, WSP 241.2.R3 (12) (Brussels, Belgium). The method is as follows: A total of 0.1 g of SAP was quantified in a 100-mesh tea bag and immersed in 100 mL of saline solution. After 30 min, centrifugation was performed at 300 G and the CRC was measured. The CRC was calculated using the following Equation (1):

(1) $$\mathrm{CRC}=\frac{\omega_{1}-\omega_{0}}{\omega_{0}}$$

where ω_1_ and ω_0_ are the weights of the swollen and dry SAP, respectively.

### 2.6. Absorbency under Load

The absorbency under load (AUL) is an absorption characteristic that measures the amount of SAP absorbing fluid under a specific load. The AUL test method was measured at a load of 0.3 psi according to EDANA, WSP 242.2.R3 (12). The method is as follows: A total of 0.9 g of SAP was quantified, placed in filter paper inside the ceramic filter, and secured in place using a glass cylinder. A 0.3 psi Teflon pressure rod was placed in SAP and a sufficient amount of saline was supplied to the Petri dish until the filter paper was soaked. The AUL was calculated using the following Equation (2):

(2) $$\mathrm{AUL}=\frac{\omega_{1}-\omega_{0}}{\omega_{0}}$$

where ω_1_ and ω_0_ are the weights of the swollen and dry SAP, respectively.

### 2.7. Permeation Time

In the SAP industry, permeability is evaluated by measuring the time it takes a certain amount of fluid to pass through the swollen gel layer. The measurement method is as follows: A total of 0.5 g of SAP was added to a glass plate of a chromatography column, 100 mL of saline was added, and swelling and precipitation were performed for 30 min. After forming the gel layer through precipitated SAP, a 0.3 psi pressure filler was installed directly above the gel layer. The time taken for 20 mL of saline to pass was measured by opening the valve at the bottom of the column.

### 2.8. Gel Strength

The gel strength of SAP was analyzed with a rotational rheometer (TA Instruments Ltd., ARES-G2, New Castle, DE, USA) with a parallel plate geometry. The plate diameter was 25 mm and plate spacing was 3 mm. The rheological measurement was performed by precipitating 0.2 g of SAP particles in 10 mL of distilled water and swelling for 30 min. After separating the gel and water, the swollen gel was placed on a parallel plate of the rheometer at 25 °C and measured. The storage modulus (G’) was recorded at a constant shear strain of 0.2% over the frequency range of 0.1–100 Hz.

## 3. Results

### 3.1. Preparation of CSAPs

**Figure 1:**
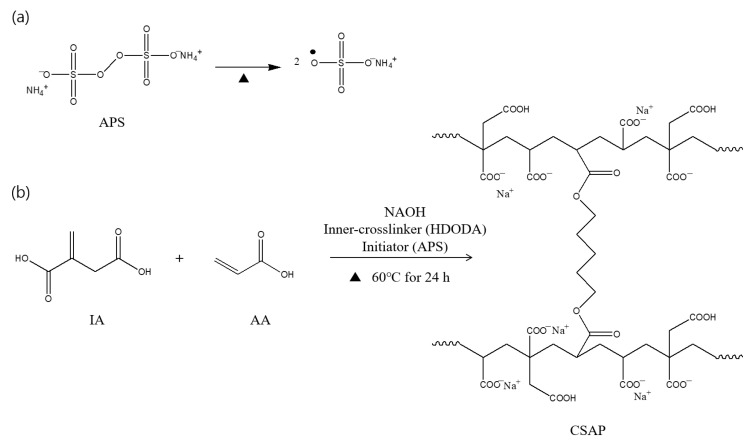
Synthetic procedures for CSAP: (**a**) thermal decomposition of ammonium persulfate, (**b**) chain propagation and crosslinking.

The poly(itaconic acid-co-acrylic acid) SAPs were prepared as described in the experimental section. These reactions and conditions are shown schematically in Figure 1. After neutralizing the carboxyl groups of IA and AA with NaOH solution, each reactant was mixed with a solution, initiation and chain growth were performed in the presence of APS, and a network structure through a crosslinking reaction was formed in the presence of HDODA. The thermal decomposition process of APS is presented in Figure 1a. The APS was initiated by generating two sulfate radical anions via the destabilization of the di-oxygen bonds between the sulfate groups [18,19]. The resulting radicals were transferred to vinyl groups contained in IA and AA of monomers to form vinyl radicals, and then a chain propagation reaction occurred. Finally, chain propagation and the reaction of the polymer and the inner-crosslinker form a crosslinked structure (Figure 1b).

### 3.2. Characterization of CSAPs

**Figure 2:**
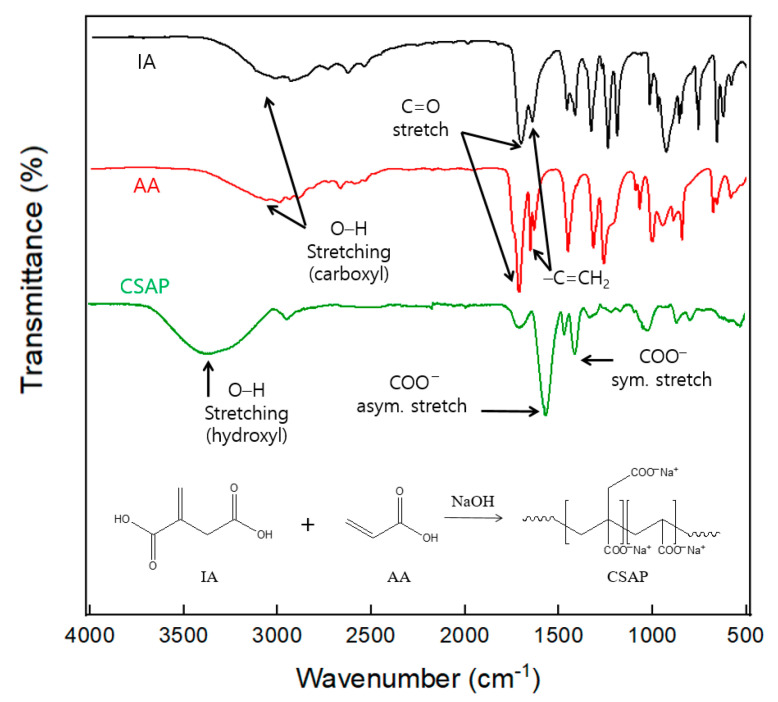
FT-IR spectra of IA, AA, and CSAP.

Figure 2 presents the FT-IR spectra of the IA, AA, and CSAP prepared with the highest feasibility in this study. The wide absorption band at approximately 2900–3350 cm^−1^ on IA and AA is attributed to −OH stretching vibrations from the carboxyl groups. The broad band observed around 3382 cm^−1^ in CSAP is the −OH stretching vibration from the hydroxyl group. The absorption peak at 2853 cm^−1^ reveals the presence of CH_2_ in the polymer backbone, while the peak at 1709 cm^−1^ is due to the C=O stretching vibrations of the carboxyl groups in IA and AA. Peaks at 911 cm^−1^ and 1638 cm^−1^ represent the C=CH_2_ vinyl groups in IA and AA, respectively. The characteristic peaks 1584 cm^−1^ and 1386 cm^−1^ in CSAP refer to carboxylate (COO^−^) ions. The carboxylate ions are generated by the neutralization of the carboxyl groups of IA and AA during the manufacturing process, and peaks of symmetry and asymmetry are presented due to the resonance structure, respectively [20]. These results confirm the presence of IA and AA in the polymer chain of the prepared CSAP and indicate a successful synthesis. The average CRC, AUL, and permeation time of CSAP before surface-crosslinking were 55.2 g/g, 10.6 g/g, and 340 s, respectively.

### 3.3. Preparation of SSAP

**Figure 3:**
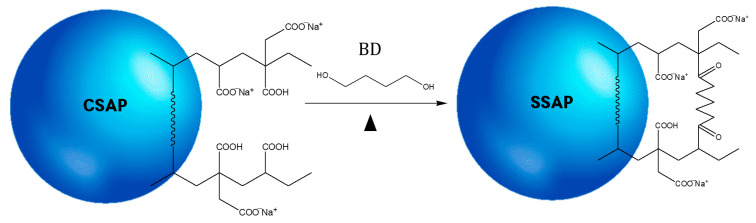
A schematic diagram of the surface-crosslinking reaction.

The surface-crosslinking reaction for CSAP is shown schematically in Figure 3. CSAP was swollen in the surface-crosslinking solution. From the short swelling time before maximum swelling, the surface-crosslinker penetrates the entire surface layer of CSAP. The core–shell structure arises due to the short absorption time and the low diffusion rate of the medium, such that the surface-crosslinker does not penetrate deeply into the CSAP network before the maximum amount of swelling is achieved [21]. The surface-crosslinking reaction was performed using 1,4-BD, which contains two hydroxyl groups (–OH) capable of an esterification reaction with the carboxyl functional group (–COOH) of the polymer chains on the surface of the CSAP particles [22].

### 3.4. The Absorption Properties via CCD of RSM

CCD was used to study the effect of the parameters CRC and AUL on absorption characteristics. The interaction mode analysis of variance was calculated using Minitab software to determine the fit of significant and nonsignificant variables, in addition to possible interactions between them. The analysis was performed using data obtained from experiments and responses to 20 runs. In the response surface regression equation, the R^2^ values are 81.70% and 81.91% for CRC and AUL, respectively, indicating moderate significance.

**Figure 4:**
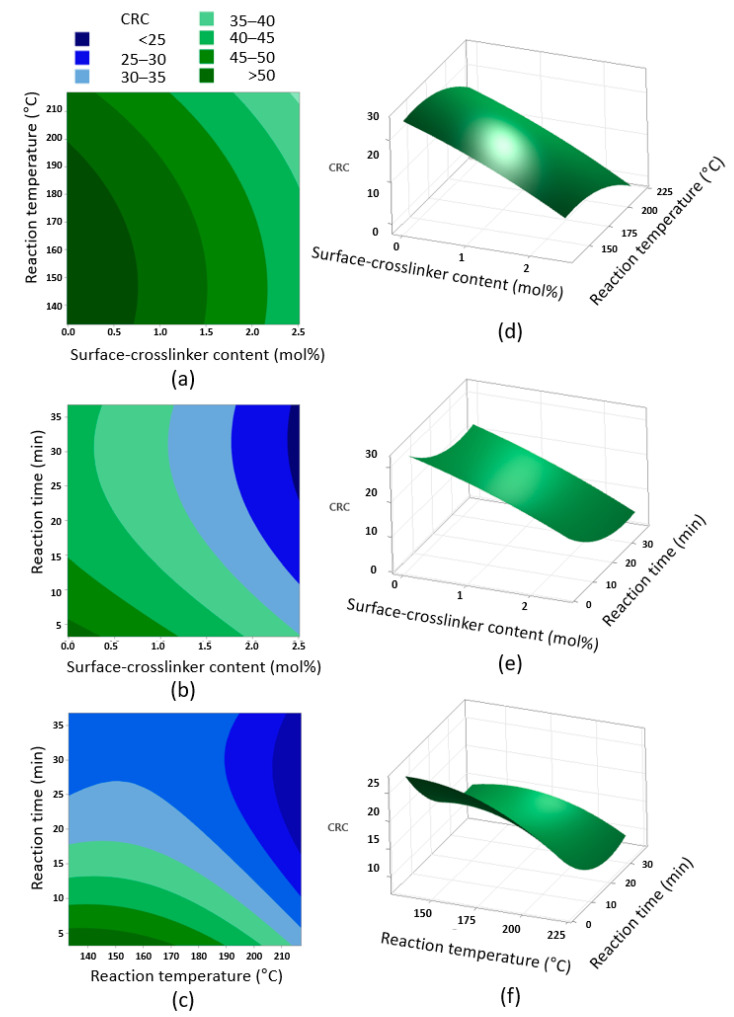
Contour and 3D surface plots via CCD of RSM between each parameter of CRC: (**a**) contour plot of CRC versus surface-crosslinker content and reaction temperature, (**b**) contour plot of CRC versus surface-crosslinker content and reaction time, (**c**) contour plot of CRC versus reaction temperature and reaction time, (**d**) 3D surface plot of CRC versus surface-crosslinker content and reaction temperature, (**e**) 3D surface plot of CRC versus surface-crosslinker content and reaction time, and (**f**) 3D surface plot of CRC versus reaction temperature and reaction time.

Contour plots with a three-dimensional (3D) response surface of the parameters for CRC are shown in Figure 4. In the contour plots shown in Figure 4a–c, a central code was applied to each excluded variable. The CRC contour plot shows clear trends. The lower the content of the BD surface-crosslinker, the shorter the reaction time: better performance was achieved at a reaction temperature of less than 170 °C. This is due to the increase in the degree of surface-crosslinking, compared with CSAP. The high crosslinking density means a decrease in the internal space of the SAP and a low swelling rate. On the other hand, the CRC decreased significantly as the reaction time increased at a high reaction temperature (above 170 °C), which is related to the thermal properties of itaconic acid-based SAP as previously studied [23]. In general, CSAPs with low crosslinking density have low thermal stability. The low gel strength resulting from low crosslinking density causes their three-dimensional network to be denatured or partially degraded from prolonged exposure to significantly high temperatures, resulting in reduced absorption properties. The response to the experiment means the SAP is unsuitable. Therefore, the conformity investigation via CCD was very meaningful.

**Figure 5:**
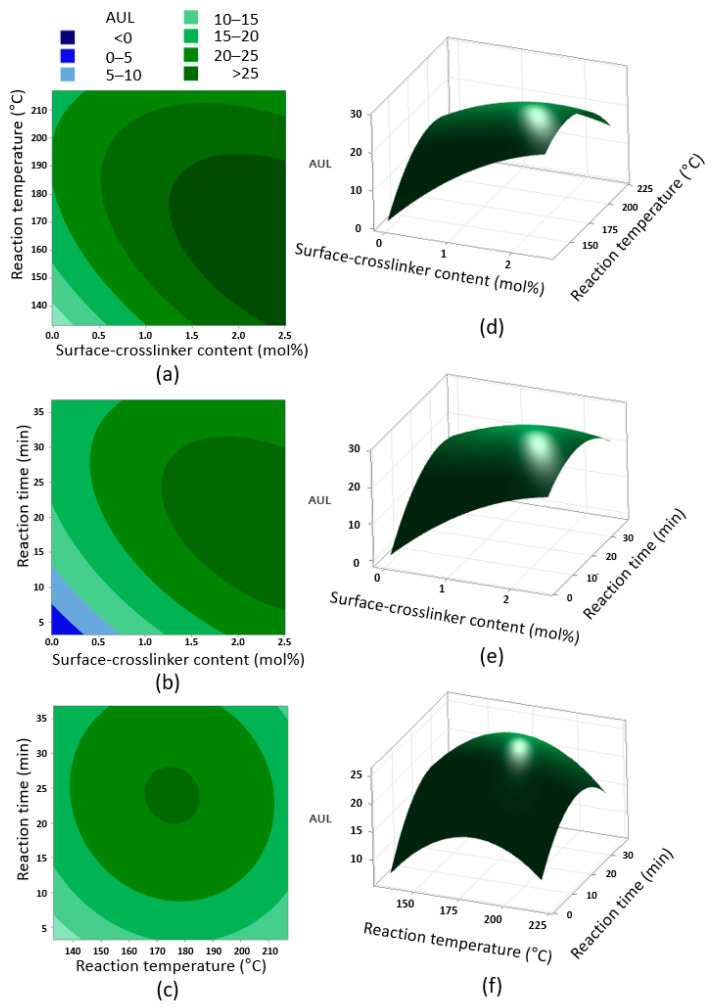
Contour and 3D surface plots via CCD of RSM between each parameter of AUL: (**a**) contour plot of AUL versus surface-crosslinker content and reaction temperature, (**b**) contour plot of AUL versus surface-crosslinker content and reaction time, (**c**) contour plot of AUL versus reaction temperature and reaction time, (**d**) 3D surface plot of AUL versus surface-crosslinker content and reaction temperature, (**e**) 3D surface plot of AUL versus surface-crosslinker content and reaction time, and (**f**) 3D surface plot of AUL versus reaction temperature and reaction time.

Contour plots with a 3D response surface of the parameters for AUL are shown in Figure 5. In the contour plots shown in Figure 5a–c, a central code was applied to the excluded variable. The trend of AUL as confirmed in the contour plots generally increases with the content of the surface-crosslinker, BD; this is the opposite of AUL as CRC increases. Reaction time and reaction temperature show high AUL characteristics in a narrow circular section (dark green) as shown in Figure 5c. The tendency according to the content of the surface-crosslinker in AUL is due to the increase in the crosslinking density, and this result can be generally explained by the improvement in the gel strength. According to the Flory–Rehner correlation, the crosslinking density is directly related to gel strength [24].

**Figure 6:**
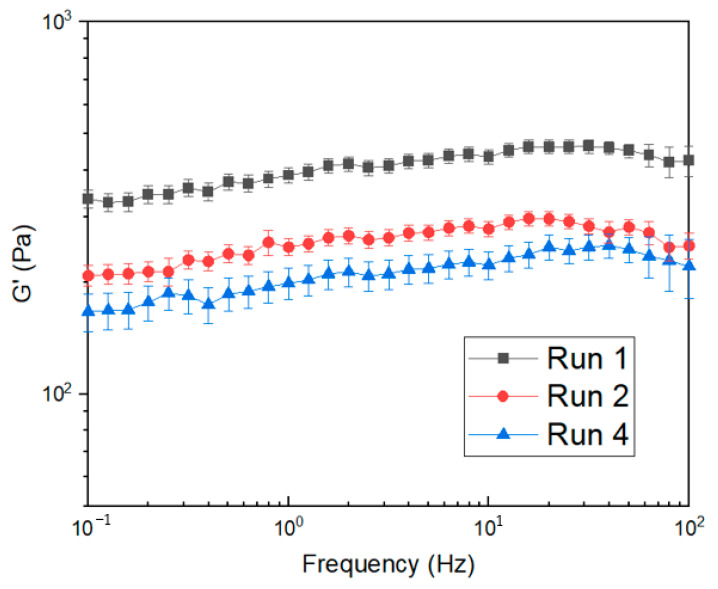
The changes in gel strength in Run 1, Run 2, and Run 4 SSAPs.

The interaction between reaction temperature and time is closely related to AUL. The optimum was exhibited at a reaction temperature of around 175 °C and a reaction time of 25 min. For a more detailed analysis of reaction temperature and time, the gel strengths of Run 1, Run 2, and Run 4 of the experimental groups in Table 1 were measured and are shown in Figure 6. In the comparison of Run 1 and 2 according to the reaction time from the central coded value of the surface-crosslinker, the gel strength decreased as the reaction temperature and reaction time increased. As shown in the CRC results, excessive reaction temperature and long reaction time adversely affect the SAP network, which is not suitable for surface-crosslinking. The standard deviation from three separate measurements on different conditions of SSAP was around 9% in Run 4 and around 6% in Run 2. The standard deviation decreased to 3% of gel strength in Run 1. This means that the bonding between BD and the polymer network was strengthened due to additional crosslinking [25].

### 3.5. The Response Optimization of CRC and AUL

The response optimization for the 20 experimental groups designed from CCD is shown in Table 2. The corresponding results set the target value for CRC for the commercialization of SAP as the minimum value of 35 g/g and the maximum value of AUL [6,23]. The confidence level in all sections was 95%, and the overall satisfaction was about 0.91, a value which is very high. The optimal surface-crosslinking process derived through CCD was approximately 2.22 mol% BD, 160 °C, and 8.7 min reaction time, with CRC and AUL at 35.0 g/g and 25.3 g/g, respectively.

Table 3 shows the results of manufacturing and measuring SAP three times based on the optimal process conditions through CCD. This validation analysis shows surprising results within the margin of error. We further investigated its liquid permeation time and recorded 46 s, which was reduced by 87% compared to CSAP. This improvement is due to the improvement in gel strength as in AUL, and has already been reported in several studies [26,27].

## 4. Conclusions

Biomass-derived superabsorbent polymer (SAP) is difficult to commercialize because it has a weakness regarding gel strength. To improve this weakness, a surface-crosslinking process was introduced. However, it was difficult to standardize the surface-crosslinking process in SAP, which provides different properties for each raw material. For the first time, we attempted to optimize the process by applying central composite design (CCD) of responsive surface methodology (RSM) to the surface-crosslinking process in the SAP industry.

The successful synthesis of CSAP utilizing itaconic acid was demonstrated through FT-IR spectroscopy, and the synthesis yield was found to be greater than 96%. SAP through the surface-crosslinking process of CSAP was manufactured through 20 experimental groups via CCD design, and three factors (surface-crosslinker content, reaction temperature, and time) were evaluated through contour plots and 3D response surface plots. The response optimization derived through Minitab software resulted in a three-factor optimal condition. The absorption characteristics of SAP from an experimental approach under optimal conditions were CRC 35.3 g/g, AUL 24.9 g/g, and permeation time 46 s. These results were derived from response optimization and values within the error range. This bio-based SAP was optimized with excellent gel strength, and the surface-crosslinking process was determined via the CCD of the RSM approach.

SAP application of the RSM-CCD modeling technique is believed to be able to present the correct surface-crosslinking process of SAP developed in various laboratories, as well as itaconic-acid-based SAP.

## Figures and Tables

**Table 1:** Experimental factors and levels in the CCD.

**Factors**		**Factor Code**	**Levels**		
			**Low** **(−1)**	**Central** **(0)**	**High** **(+1)**
**Surface-Crosslinker (mol%)**		**A**	0.50	1.25	2.00
**Reaction Temperature (°C)**		**B**	150	175	200
**Reaction Time (min)**		**C**	10	20	30
**Run No.**	**Coded Factors**				
	**A**	**B**		**C**	
1	1.25	175		20	
2	1.25	175		36	
3	1.25	175		20	
4	1.25	217		20	
5	1.25	175		20	
6	2.00	150		10	
7	0.50	150		30	
8	0.50	200		10	
9	1.25	175		20	
10	2.00	150		30	
11	0.50	200		30	
12	0	175		20	
13	2.00	200		10	
14	1.25	175		20	
15	2.00	200		30	
16	1.25	132		20	
17	1.25	175		3	
18	0.50	150		10	
19	1.25	175		20	
20	2.51	175		20	

**Table 2:** Optimum conditions derived by CCD of RSM design.

Optimal Conditions			Absorption Properties		Overall Satisfaction
BD (mol%)	Reaction Temperature (°C)	Reaction Time (min)	CRC (g/g)	AUL (g/g)	
2.2216	160.0630	8.6614	34.9993	25.3707	0.9125

**Table 3:** Reproducibility experiments under optimal conditions.

Run	CRC (g/g)	AUL (g/g)	Permeation Time (s)
SSAP optimum	35.3 ± 0.4	24.9 ± 0.7	46 ± 3

## Data Availability

Not applicable.
